# Sciatica caused by lumbar epidural gas

**DOI:** 10.11604/pamj.2014.18.162.1354

**Published:** 2014-06-19

**Authors:** Hatim Belfquih, Brahim El Mostarchid, Ali Akhaddar, Miloudi Gazzaz, Mohammed Boucetta

**Affiliations:** 1 Department of Neurosurgery, Mohammed V Military Teaching Hospital, Rabat, Morocco

**Keywords:** Epidural gas, intradiscal vacuum phenomenon, lumbar spine, sciatica

## Abstract

Gas production as a part of disc degeneration can occur but rarely causes nerve compression syndromes. The clinical features are similar to those of common sciatica. CT is very useful in the detection of epidural gas accumulation and nerve root compression. We report a case of symptomatic epidural gas accumulation originating from vacuum phenomenon in the intervertebral disc, causing lumbo-sacral radiculopathy. A 45-year-old woman suffered from sciatica for 9 months. The condition worsened in recent days. Computed tomography (CT) demonstrated intradiscal vacuum phenomenon, and accumulation of gas in the lumbar epidural space compressing the dural sac and S1 nerve root. After evacuation of the gas, her pain resolved without recurrence.

## Introduction

Intraspinal gas is very rare and it was first reported in 1980 by Gulati and Weinstein [1]. Intradiscal gas (vacuum phenomenon), which is suggested to be origin of intraspinal gas,has been observed since 1910 [2], and it has been observed in radiographs since 1942 [3]. Gas within the spine can produce sciatica via two pathophysiological mechanisms: gas can accumulate either within a herniated disk or within the epidural space in the absence of disk herniation [4]. The clinical features are similar to those of common sciatica. CT is very useful in the detection of epidural gas accumulation and nerve root compression [5]. We report these cases of sciatica caused by gas in the epidural space and discuss the diagnosis and management of this rare cause of sciatica.

## Patient and observation

**Figure 1:**
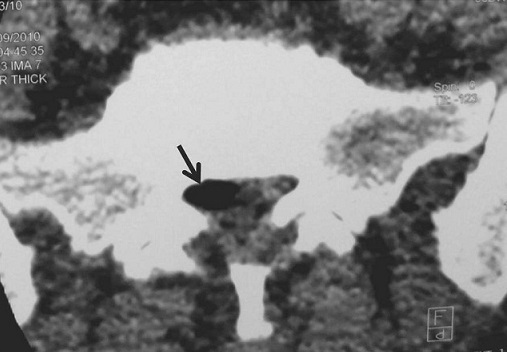
Computed tomography (CT) axial view showed a 10 mm gas bubble (arrow) in the right anterolateral portion of the epidural space at L5-S1 level compressing the dural sac and right S1 nerve root

**Figure 2:**
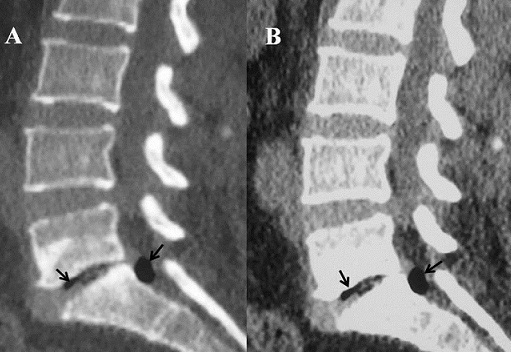
CT sagittal reconstruction (A, B) revealed vacuum phenomenon in intervertebral disc (arrow) with moderate disc protrusion at this level

A 45- year-old previously healthy woman presented with a 9- months history of right radicular leg pain. No precipitating factor to the pain was identified, and there was no history of excessive motion or sports before the onset of clinical symptoms. The pain had become significantly worse during walking in the several days before presentation. On admission; physical examination showed a positive straight leg raising at 65° without motor or sensitive deficit. Sphincter function and the left side were normal. CT showed vacuum phenomenon with moderat disc protrusion at L5-S1 space, and gas bubble in the right anterolateral portion of the epidural space at this level. Epidural gas accumulation was compressing the dural sac and right S1 nerve root (Figure 1, Figure 2). The pain was severe and failed to respond to conservative therapy including analgesics, anti-inflammatory and muscle relaxant drugs. An ipsilateral L5-S1 interlaminar approach was performed with enlargement of the lateral recess. The S1 root appeared swollen and compressed by the adjacent pseudo-cyst that was removed. Histological study revealed no specific fibrous tissue. One year after this operation, the patient remains free of pain.

## Discussion

The vacuum phenomenon is the creation of an air space in a degenerated intervertebral disc as a result of movement of the spine, especially extension and traction [3]. Trauma, pyogenic infections, pneumothorax, and iatrogenic instrumentation such as percutaneous vertebroplasty and spinal surgery are the other less-common underlying mechanisms [1]. The created space fills up with gas containing nitrogen, which comes from gases dissolved in the extracellular fluid that diffuse into areas of subatmospheric pressure. In patients with degenerative disc disease, the nitrogen is liberated in the disc fissures and cannot be reabsorbed or replaced by liquid because the degenerated disc is avascular [5]. The vacuum phenomenon of the disc is seen in as many as 50% of patients over 40 years of age [6]. If the anulus fibrosus ruptures, this air is released and collects in the epidural space. Accumulation of gas in the epidural space is rare finding and an unusual cause of radiculopathy. Gas within the spine can produce sciatica via two pathophysiological mechanisms: gas can accumulate either within a herniated disc or within the epidural space in the absence of disc herniation [4]. The clinical features are similar to those of common sciatica, with mechanical monoradicular pain, antalgic posture, and a positive straight leg-raising test [5].

CT is the investigation of choice for the diagnosis. The scans not only show that the collection within the spinal canal is composed of gas but also provide useful information on the condition of the disc and of the rest of the lumbar spine. The typical findings include degenerative disc disease with central vacuum phenomenon and, at the same level, a collection of epidural gas in contact with the nerve root corresponding to the distribution of the pain. This last point is important because epidural gas is sometimes present in asymptomatic patients. The gas collection can range in size from a few millimeters to 1 centimeter and in density from - 200 to - 900 Hounsfield units. Rim enhancement can be seen. MRI yields similar findings, with low signal on T1- and T2-weighted images and postgadolinium rim enhancement [1]. Gas in the epidural space may be absorbed spontaneously. Therefore, in patients with gas-related neurologic symptoms, conservative treatment with nonsteroid anti-inflammatory drugs and muscle relaxants should be the first choice. Percutaneous, intravenous, and oral steroids have also been reported, in combination with epidural glucocorticoid injections if needed as treatment options. Aspiration of the gas collection under fluoroscopic guidance has been used, but when the procedure induced pain relief, this effect lasted 6 months at the most [7, 8]. Surgery is in order in patients who fail to respond to conservative therapy. Because the gas is produced within the disk, the procedure consists not only of removing the gas collection but also in curetting the disk space.

## Conclusion

Epidural gas collection when located near a nerve root may exercise a compression phenomenon that may cause symptoms such as low back pain and radiculopathy. CT is the best imaging method of the evaluation of the gas in the lumbar spine due to the heavily negative Hounsfield units of the gas. Surgery is performed in patients who fail to respond to conservative therapy, the procedure consists not only of removing the gas collection but also in curetting the disk space.
